# The age of chronic diseases onset – the results of the longitudinal study

**DOI:** 10.1093/eurpub/ckac131.126

**Published:** 2022-10-25

**Authors:** D Skybova, H Slachtova, H Tomaskova, J Klanova, R Madar

**Affiliations:** 1 Faculty of Medicine, University of Ostrava, Ostrava, Czechia; 2 Centre for Epidemiological Research, University of Ostrava, Ostrava, Czechia; 3 Department of Occupational Medicine, Public Health Institute, Ostrava, Czechia; 4 Centre RECETOX, Faculty of Science, Masaryk University, Brno, Czechia

## Abstract

**Background:**

A current aging of the European population call questions into how to protect a high quality of health in the elderly which was the reason to establish the longitudinal HAIE Project. As the baseline information, the longitudinal ELSPAC study results were used. The aim of the analysis was to learn a life-course-dependent risk factors of the onset chronic diseases in middle-aged population.

**Methods:**

The cohort includes data on both parents. Their number decreased during the follow up from 4,500 to about 1,000 parents. The selected questionnaire data of the cohort gained at consecutive intervals during the years 1990-2010 were analysed descriptively, according the distribution of data by respective statistical methods and survival analysis. Differences between curves were tested by log-rank tests (significance level of 5%). The SW Stata v.15 was used.

**Results:**

The mean age of mothers (N = 823) at study entry was 25.0 years (SD = 4.86), the age of fathers (N = 385) 28.8 years (SD = 6.05). Good self-reported health has decreased continually over time from more than 80% in both parents aged 30 to about 50% in the age of mid-forties. Most parents suffered from back pain, hypertension, and joint pain. The prevalence of diseases has raised over time. Problems with hypertension begin in women from the age of 38 (39 in men) and there was no statistically significant difference by sex (p = 0.265). Survival analysis found a significant difference by sex for depression (p < 0.001) and physician-confirmed back pain (p < 0.001), which affect parents from 35 years (significantly more in women than men).

**Conclusions:**

The processed analysis will refine the findings of the longitudinal Project HAIE (CZ.02.1.01/0.0/0.0/16_019/0000798) and subsequent projects monitoring the factors influencing healthy aging. The (C)ELSPAC studies are supported by the projects RECETOX RI (No LM2018121) and CETOCOEN EXCELLENCE (No CZ.02.1.01/0.0/0.0/17_043/0009632).

**Key messages:**

• About 50% of the mid-forties aged population did not declare good self-reported health. They suffered mostly from back pain, hypertension, and joint pain. The diseases prevalence has raised over time.

• The onset of hypertension begin from the age of 38 (39) with no difference by sex, depression and physician-confirmed back pain start from 35 years of age, significantly more in women than men.

